# The integration of mental health care act in primary care: an audit of the use of mental health care act forms for patients´ admission and the effect of continuing medical education on health professionals´ performance of usage, based on Letsholathebe II Memorial Hospital´s experience, in Maun, Botswana

**DOI:** 10.11604/pamj.2021.40.49.26114

**Published:** 2021-09-20

**Authors:** Deogratias Ongona Mbuka, Stephane Tshitenge, Adekunle John Ogunjumo

**Affiliations:** 1 Department of Family Medicine, University of Botswana, Maun, Botswana,; 2 Department of Family Medicine, University of Botswana, Mahalapye, Botswana

**Keywords:** Mental health care act (MHCA), primary health care (PHC), voluntary admission, Botswana, LIIMH, mental health

## Abstract

**Introduction:**

despite the adoption of mental disorders act in 1972, the use of required mental health care act (MHCA) forms during admission of patients with mental illnesses remained below the legal expectation in the Maun District Hospital. This study audited Letsholathebe II Memorial Hospital (LIIMH) professionals´ usage of MHCA forms.

**Methods:**

this was a quasi-experimental study that audited files of patients admitted with mental illnesses, before, three and six months after a continuing medical education (CME). Cochran Q, McNemar symmetry Chi-square were used for comparison of performance.

**Results:**

of the 239 eligible files, we accessed 235 (98.3%). About two in ten (n=36/235, 15.3%) MHCA forms were not used in combination with required forms. The quasi-majority of MHCA forms set used, aligned with involuntary admission (n=134/137, 97.8%). Required admission MHCA forms significantly increased from nil before continuing medical education (CME-0), to 64.6% (n=51/79) at CME-3 and 77% (n=59/77) at CME-6 (p<0.001). However, there was no statistical difference between the last two periods (64.6% vs 77%, p=0.164). Voluntary admission remained below 13% (n=10/79). Only six types of MHCA forms were used during this study.

**Conclusion:**

there was no adequate use of required MHCA forms at LIIMH before CME. Thereafter, the proportion of adequate use increased from period CME-0 to the periods CME-3 and CME-6. However, there was no difference in proportion between the last two periods. We recommend an effective and regular CME twice a year for health professionals on selected MHCA forms.

## Introduction

The global prevalence of mental and neurological disorders represents up to 48.6% in life time and about 29.1% in a twelve months´ time, with the three quarter of the burden occurring in the developing countries [1]. According to the World Health Organization (WHO) Atlas, there is evidence of a global increase in mental health issues with uneven distribution of resources across the regions and within countries with inadequate resources [2]. Mental health is an issue of public health concern in primary care settings in Africa and requires adequate attention on different aspects, one of which being resource allocation [3]. For illustration, about two to three in ten patients who visited primary health care (PHC) out-patients in Uganda had mental health issues [4,5]. A South African study reported similar mental health prevalence in PHC [4], although reports from Ghana mentioned a lesser percentage [6]. Like other commonwealth countries, Botswana uses the legal framework of rules and regulations which is the mental health care act (MHCA) to protect the right of patients with mental illnesses as well as that of health professionals who are providing care [7]. In the Botswana MHCA, depending on whether the admission is voluntary or not, forms are available to regulate types and duration of admission, as well as possibly a transfer to a mental hospital by the attending health professionals [8]. There are 17 types of forms available for use under MCHA bearing form number one to seventeen [8]. As a rule, forms are used in combination rather than singly. Hence, under involuntary admission, an urgent application (form 6) and a medical certificate (form 2) are to be used. Under voluntary admission, different stakeholders (applicant and medical practitioner) fill form 14 (voluntary admission) and form 16 (medical recommendation on admission).

The implementation of MHCA in PHC setup requires a systematic approach [9]. Studies suggested that to overcome shortcomings in implementing appropriate MHCA in PHC setting, health professionals should be trained in mental health issues and MHCA [10]. Unfortunately, this may not be the case to all health professionals in Botswana. Continuing medical education (CME) done as in-service training is a strategic way for health professionals to acquire skills, improve or maintain competencies for a quality health system delivery [11]. To be successful a CME must really be continuous and regular [12]. A seminar is one of the effective teaching and learning methods in CME. It is based on a lecture and demonstration format in which visuals materials, interactive tools and demonstrations are combined [13, 14].

A seminar addresses both low intellectual capacities outcomes (knowledge, comprehension, and application) and high intellectual capacity outcomes (analysis, synthesis, and evaluation). A seminar is also linked to attitudes outcomes (aware and habit) and development of the ability to communicate [15]. Using MHCA forms by both health professionals and other officers who reinforce mental health policies has been reported to be variables in terms of detailed documentation, appropriateness of usage and compliance. For example, in Australia over 10% of mental health forms failed to meet MHCA requirement of involuntary admission, with consequent unlawful detention [16]. Also, MHCA certificates errors of documentation by physicians were reported in varied proportion from 11% to 19%, implying the magnitude of unlawful detention by physicians in Canada [17]. Reports on usage of MHCA forms, their types, and their appropriateness in our setting, could not be accessed. Knowing the proportion, types and how MHCA forms are used in our setting could help deciding on relevant forms for use in Botswana PHC in line with the decision of implementing the MHCA in the PHC setting. This study intended to audit the use of MHCA forms by health professionals before, three and six months after a CME in Letsholathebe Memorial Hospital (LIIMH), a district hospital, in Botswana. Researchers hypothesized that there will be an improvement in proportion of use of MHCA forms after the CME.

## Methods

**Study design and period:** this is a quasi-experimental study auditing the use of MHCA forms on admission of patients with mental illnesses before, three months and six months after a CME seminar session. The study period was from March 2015 to February 2016.

**Setting:** Letsholathebe II Memorial Hospital is a district hospital in the Ngami Health District North-Western of Botswana. It has a 300-bed capacity, of which the psychiatric unit has 15 beds that can be extended to 26 floor beds capacity. Letsholathebe II Memorial Hospital is one of the training sites of family medicine, University of Botswana (UB). The psychiatric unit is run by nine nurses supported by a psychiatrist and a medical officer when available from the doctors´ pools in the hospital. Patients´ physical records of MHCA forms and files are kept in psychiatric unit during admission and a short period after the patient has been discharged before being sent to and kept at the hospital records unit. Admissions of patients with mental illness are done from outpatients and emergency department (ED) to the psychiatric unit which admits on average 25 patients in a month. Medical officers admit those with conditions that require medical care to medical wards for stabilization prior to the admission to the psychiatric unit. To admit patients with mental illness, they are expected to fill appropriate MHCA forms.

They ask the patient´s near relative or any other person who believes that the patient is mentally disordered or defective to fill specific MHCA forms in support to the applied admission. In case of patient´s voluntary admission, a set of MHCA forms that are filled includes a form 14 ( voluntary own admission application), filled by the patient above 16-year-old applying for own admission as a voluntary patient or form 15 used for patients under 16 years, while doctors fill the form 16 (medical recommendation). In involuntary admission, the set of MHCA forms that are filled include form 6 (urgent application), filled by near relative or any other person who has witnessed within the last 48 hours the patient´s mental state with specific behavioural changes, while form 2 (medical practitioner certificate) is filled by the doctors to testify patient´s mental state [8]. Admission MHCA forms are valid for 14 days. If there is need for an admission of more than 14 days, a near relative or any other person who has witnessed the patient´s mental state with specific behavioural changes within the last 14 days is required to fill a form 1 (application for reception order) before the expiration of the initial 14 days of admission. Form 1 and form 2 are then sent to the district commissioner who fills form 4 (reception order) which is valid for 30 days [8,18,19]. Form 4 can only be renewed twice. In case of admission that requires more than 90 days, a transfer to mental health institution through appropriate procedure is considered. For each admission, a minimum of two valid forms are required.

**Study population and sampling:** discharged patients´ records from March 2015 to February 2016 constituted our study population. We took all records falling within the selected three consecutive months as we anticipated that the study population would not be very large (about 75 accessible records). Hence we reviewed three months records before the CME and the subsequent three consecutive months at three and six months after the CME, eight months following the CME, from 1^st^ March 2016 to 31^st^ May 2016.

**Procedure and data collection:** the team that trained LIIMH healthcare professionals comprised nurses working at LIIMH psychiatric unit, a psychiatrist and the principal researcher, who is a family physician, academic staff from the University of Botswana (UB). Letsholathebe II Memorial Hospital healthcare professionals involved in the care of patients with mental illnesses outpatients, emergency unit, medical ward and psychiatric unit were sensitised in their units every morning during handover meeting for 2 weeks following the CME. In total, 20 medical officers and 25 nurses from the hospital were trained; representing more than 3/4 of the targeted staff involved in the management of patients with mental illnesses. The 2-hour CME-seminar that took place on 24^th^, June, 2015, consisted of an hour didactic on the mental health act rational and presentation of the mental health forms followed by one hour interactive scenarios based illustration on the choice of the appropriate mental MHCA forms and steps in filling of forms by participants. The team reviewed the psychiatric unit patients´ register to retrieve patients´ files from psychiatric and hospital record/archive (HR/A) units of the three consecutive months prior to the CME (CME-0) from 1^st^ July to 30^th^ September 2015, and six months after CME (CME-6) from 1^st^ December 2015 to 28^th^ February 2016. Members of the team got discharged list of patients the from psychiatric ward registry. The actual retrieval of files (patients´ records) happened in the HR/A unit with an established list from the psychiatric ward registry. They retrieved data from files in chronological order of 5 to 15 files at a time depending on the officer on duty at HR/A, then returned files to HR/A before a subsequent lot was collected.

**Data analysis:** a panel of two experts, the principal researcher, and research assistant with expertise in the use of MHCA forms read through all files of the selected study periods. They had to make a consensus from the patient´s history and when necessary, they got clarifications from psychiatric nurses as to whether MHCA Forms were filled appropriately. The designed check-list that meant to assess recommended standard procedures for MHCA form according to the mental health act process was used [8,20]. The check-list gathered information such as patient´s characteristics, duration of admission, evidence of types of mental health act forms used in patients´ records, the question on the appropriateness of forms used in patient´s files. If the consensus was not met, the team put the file aside. Data were summarized using frequency, mean (± sd) or median (± IQR) where appropriate. Tables and figures were also used to summarize the information. The proportion of MHCA forms used, were assessed through the presence of admissions forms namely: urgent application and medical certificate, voluntary admission and medical recommendation, or reception order with a medical certificate. As we compared the performance of same LIIMH staff in the utilization of MHCA forms, their appropriateness of usage between two and three periods, namely periods CME-0, CME-3 or CME-6, we used Cochran Q test and McNemar Symmetry chi-squared in post hoc analysis to determine significant difference between two compared periods. The level of significance of 0.05 was used, while the Bonferroni correction cutting point of significance was 0.017 (α=0.05/m=3). The statistical package for the social sciences (SPSS) version 25 software was used.

**Ethical considerations:** researcher got the permission from the Ngami DHMT Institutional Review Board with number: M6/50/12 I. Patients and health professionals involved in the use of different files were kept confidential by the team involved in the data collection and analysis. A shared (joint) responsibility on the findings of this study was agreed upon in the psychiatric unit.

## Results

Out of the 239 eligible files from the psychiatric ward registry, 235 (98.3%) files were accessed from HR/A. Among those 235 accessed files, 79 (33.6%) were of the period CME-0, 79 (33.6%) of the period CME-3, while 77 (32.8%) were of the period CME-6. Three-quarters (median: 29, (IQR±13) of admitted patients were of age 42 years or less. About two-third (n=151, 64.3%) of the accessed files were from male patients, while 84 (35.7%) files were from female patients. Three-quarters (n=181, 75%) of patients admitted to psychiatric ward spent up to 14 days (Table 1). In total, 36 forms were not adequately used in combination with other forms as required; this represented about two in ten (n=36/235, 15.3%) of accessed files. Six in ten (n=22/36, 61.1%) of these used alone forms were from the CME-3 period. Medical practitioner certificate (form 2) and urgent application (form 6) were the most MHCA forms singly used, with 48.7% (n=19/36) for form 2 and 30.7% (n=12/36) for form 6. If no single voluntary admission form for less than 16-year-old was filled, one of these forms was filled for patients of more than 16-year-old. Of all the MHCA forms set used for patients´ admission, only three set of forms out of 137 records (n=3/137, 2.2%) represented voluntary admission modality. Most of the combined forms were from the period CME-3 (n=64, 46.7%) and the period CME-6 (n=70, 51%). The majority of MHCA forms set used were aligned with the involuntary admission modality of patient (n=134/137, 97.8%). Among those, about seven in 10 files (n=95, 69.3%) were a combination of urgent application (form 6) with medical practitioner certificate (form 2) (Table 2).

**Figure 1:**
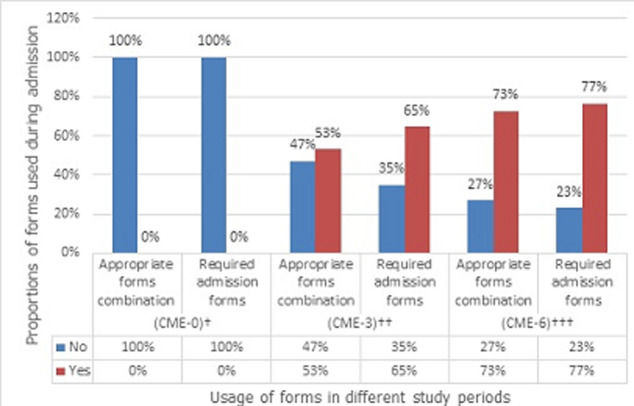
required admission forms and appropriate forms combination usages, before and after a CME at Lethsolathebe Memorial Hospital (March 2015 to February 2016)

The proportion of required two MHCA forms on admission for voluntary and involuntary modality in combination of usage increased from nil in the period CME-0, to 65% (n=51/79) during the period CME-3 and 77% (n=59/77) during the period CME-6 (Figure 1). There was a significant difference between these three proportions (p<0.001). However, the difference between the proportion of MHCA admission forms for voluntary and involuntary modality in a combination of usage, was not significant between the period CME-3 and the period CME-6 (65% vs 77%, p=0.164). The appropriate combination of mental health forms during admission increased from nil at the period CME-0 to 53% (n=42/79) at the period CME-3 and to 73% (n=56/77) at the period CME-6 (Figure 1). These differences across the three periods were significant (p<0.001). There was also a significant difference in proportion of these combinations of MHCA forms between the period CME-3 and the period CME-6 (53% vs 73%, p=0.02).

**Table 1:** characteristics of accessed files of patients from the Lethsolathebe Memorial Hospital psychiatric ward registry (March 2015 to February 2016)

		Accessed files CME-0 ✝ n (%)	Accessed files CME-3 ✝✝ n (%)	Accessed files CME-6 ✝✝✝ n (%)	Total n (%)
**Expected registry files**		79 (33.1%)	81 (33.9%)	79 (33.1%)	239 (100%)
**Missing files**		-	2 (0.85%)	2 (0.85%)	4 (1.7%)
**Accessed files**		79 (33.6%)	79 (33.6%)	77 (32.8%)	235 (98.3%)
**Age: median (± IQR)**		29 (±13)	30 (±12)	29 (±12)	29 (±13)
**Sex**	**Male**	51 (64.6%)	55 (69.6%)	45 (58.4%)	151 (64.3%)
	**Female**	28 (35.4%)	24 (30.4%)	32 (41.6%)	84 (35.7%)
**Duration of admitted**	**Less than 14 days**	65 (82.3 %%)	54 (68.4%)	62 (80.5%)	181 (77%)
	**More than 14 days**	14 (17.7%)	25 (31.6%)	15 (19.5%)	54 (23%)
**Total n (%)**		79 (33.6)	79 (33.6)	77(32.8)	235 (100%)

n: number; IQR: interquartile range; ✝: three consecutive months prior to CME; ✝✝: three consecutive months from three months after CME; ✝✝✝: three consecutive months from six months after CME

**Table 2:** proportion of voluntary and involuntary MHCA Forms usage on patient's admission before and after CME at LIIMH (March 2015 to February 2016)

MHCA forms		Accessed files CME-0✝ n (%)	Accessed files CME-3✝✝n (%)	Accessed files CME-6✝✝✝ n (%)	Total n (%)
Voluntary admission	Combine Form 14 or 15 and Form 16	-	1 (1.3%)	2 (2.5%)	3 (2.2%)
Involuntary admission	Combine Form 2 and Form 6	-	42(53.2%)	53 (68.8%)	95 (69.3%)
	Form 4 usage on admission	3 (3.8%)	12(15.2%)	8 (10.4%)	23 (16.8%)
	Combined Form 2 and Form 4	-	9 (11.4%)	7 (9%)	16 (11.7%)
Total n (%)		3 (2.2%)	64 (46.7%)	70 (51%)	137 (100%)

MHCA: mental health care act; n: number; ✝: three consecutive months prior to CME; ✝✝: three consecutive months from three months after CME; ✝✝✝: three consecutive months from six months after CME

**Figure 2:**
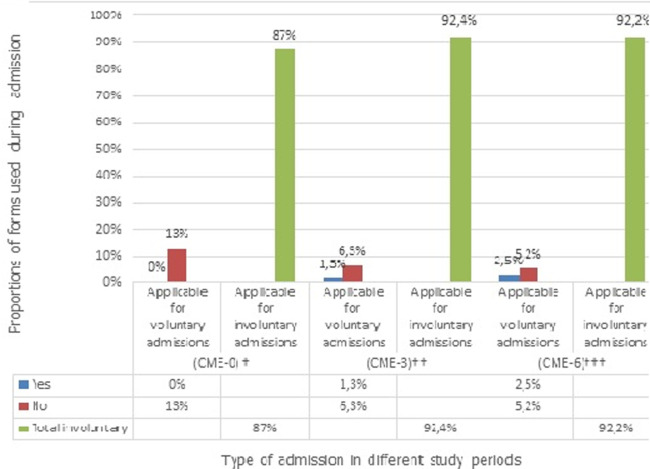
voluntary and involuntary admission forms usage before and after CME at LIIMH (March 2015 to February 2016)

Like the use of required two admission forms and the appropriate combination of MHCA forms set usage, the use of urgent application forms in combination with medical certificate forms during patients´ admission improved from nil at period CME-0 to 52% (n=41/79) at period CME-3 and 69% (n=53/77) at period CME-6. These differences were significant across the three periods (p<0.001). However the difference between the proportions of these MHCA forms set usage at periods CME-3 and CME-6 were not significant (52% vs 69%, p=0.07). Across the three periods the proportion of involuntary admission was similar and most of the time applicable in 87% (n=69/79) at period CME-0, 92.4% (n=73/79) at period CME-3 and 92.2% (n=71/77) at period CME-6 (87% vs 92.4% vs 92.2%, p=0.5) (Figure 1). However, the proportion of files requiring voluntary admission remained low with 13% (n=10/79) at CME-0, 7.6% (n=6/79) at CME-3 and 7.5% (n=6/77) at CME-6. There was no record of voluntary admission form in CME-0, while 1.3% (n=1/79) of filled voluntary admission forms was found in CME-3 and only 2.5% (n=2/77) in CME-6 (Figure 2). Also the proportion of reception orders used during patient´s admission remained low for the period before and after the CME. Only 3.8% (n=3/79) of forms were found for the period before the CME, while 15% (n=12/79) of forms were for the period CME-3 and 10% (n=8/77) forms for the period CME-6. No difference was observed between these proportions (3.79% vs 15% vs 10%, p=0.062). Throughout the three periods of this study, the timely use of reception order and its use for patient´s admission remained below the 15% (n=12/79).

## Discussion

This study was about auditing health professionals´ use of MHCA forms in three periods namely CME-0, CME-3, and CME-6 at LIIMH with hypothesis that there will be an improvement of the use of MHCA forms three and six months after the CME. The present study indicates that three-quarters of admitted patients were young with age below 42 years. Although the treatment of psychiatric disorders may occur later, the first onset of mental disorders usually occurs in childhood or adolescence [21]. This may explain the young age of patients admitted to the LIIMH psychiatric unit. In this study about two-third (64.3%) of the accessed files were from male patients. Authors such as Tadesse *et al*. and Scott *et al*. reported a similar proportion as about six psychiatric patients in 10 were males [22]. The present study found that at period CME-0, the use of required MHCA forms that guarantee lawful admissions of the patients in the psychiatric unit was exceptionally low at LIIMH. Of the 36 files that had a single MHCA form used, only one file had a MHCA form 4 (reception order) at period CME-0. The proportion of form 4 used during patient´s admission remained low for the period before and after the CME, even though the number of form 4 seemed to have increased to 22 (61.1%) at period CME-3 and 13 (36.1%) files at period CME-6. This trend of low usage of form 4 could be the result of the CME training on required admission forms, considering that in the majority of the emergency admission, the initiation of the process and the actual use of reception order might be long and unpractical compared to other MHCA forms. The average shorter hospital stays noted with most patients with no need for an extension of the initial admission MHCA forms validity may have contributed to the low proportion of form 4 usage. The review of any combined MHCA forms noted only 2.2% of the total reviewed 137 files at CME-0. However, this proportion increased to 46.7% at period CME-3 and to 51% of reviewed files at period CME-6. These differences were significant across the three study periods (p<0.001). However the present study did not observe a significant difference in proportion of use between periods CME-3 and CME-6 (p=0.164). The appropriate combination of MHCA forms during admission increased from nil at the period CME-0 to 53.2% at the period CME-3 and to 73% at the period CME-6 (p<0.001). The comparison in proportion of MHCA forms combination used in the two periods, CME-3 and CME-6 showed a significant increase of use (p=0.02). The implication of these findings is that the CME improved participants´ attitudes and skills in the use of MHCA forms only during the first three months after training. Six months after the training health professionals at LIIMH seemed not to have improved their performance of MHCA forms usage gained by three months. Very often modes of delivery CME are based on the available time for training, available budget, and on learning outcomes. With this approach, health professionals taking the CME may not have achieved sustainable knowledge, attitude, and skills of the taught topic.

The performances in MHCA are reported as poor in both developing and developed countries. For instance, it was reported that in Australia only 40% of a data set of 2941 MHCA forms filled by doctors complied to the required legal criterion for detention under the involuntary admission act [23]. The absence of the usage of legal forms during involuntary admission of patients before the CME in this study could be of serious concern for both the patients´ rights and for impeding litigation to the health facility, which should always be avoided as part of good clinical practice. In this study, most patients with mental illness were eligible for involuntary admission with 87.3% forms at period CME-0, 98.6% at period CME-3 and 97.3% at period CME-6. Very few studies published on the proportion of involuntary admission. In available data, the proportion of involuntary admission of patients with mental illness seemed to be about 70% [24], which is below the report of this study. Regarding the length of stay in the hospital, three-quarters (75%) of patients admitted to the psychiatric ward spent up to 14 days. Worldwide, most of general acute psychiatric cases admissions in non-psychiatric referral institutions last less than two weeks [25]. South African mental health regulation is that 72 hours patients´ assessment is observed in a non-psychiatric unit before an extension of the involuntary admission or a conversion of the involuntary admission to a voluntary with possible referral to a psychiatric facility [26,27]. In Botswana, primary hospitals have dedicated wards for lawful admission of patients with mental illnesses, provided the admission is done using the required set of forms under voluntary or involuntary admission. Reported 14 days of hospital stay by most records in this study has an implication on the type and regularity of renewal of MHCA forms in our setting. Considering that majority of admission MHCA forms are valid for 14 days, most of the time there may be no need to extend patients´ hospital length of stay in PHC setting. However, for few patients who were found to have hospital stay of more than 14 days, a reception order (form 4) is the next MHCA form to be considered for use.

This is because it allows a number of extensions of up to 30 days each, to a maximum of two renewals plus the first-time application which makes the maximal total extension to 90 days. Hence the optimisation of the use of these essentials forms will contribute to the implementation of the integration of MHCA in PHC setting. These forms are known as the urgent application form and the voluntary admission form to be used with medical certificates and medical recommendations respectively, while the reception order will require a reception order application to be accompanied with a medical certificate. In support to the above trend of hospital stay it is known that substance abuses are associated with a shorter length of stay, while mood or psychotic disorders are associated with a long stay in hospital. In addition, female patients with mental illness have the tendency to be admitted longer than male patients [28]. The present study did not look at the proportion of acute versus chronic psychiatric conditions, male versus female length of admission, to compare such findings with the local LIIMH trend in that regard. Assessing a possible association between the types of psychiatric disorders gender and length would have been enlightening. The use of patients’ records in this study with possible poor documentation issues may have had an impact on current findings. Current applied audit approach findings will be specific to LIIMH and will not consequently be generalized.

To achieve a behaviour change in clinical practice as a result of any CME, it is important to be mindful of the alignment between the methods of delivery of teaching for a desire outcome [11,13,15]. In our case with the teaching of MHCA, we expected participants would be able to use the MHCA because of the CME. It is also important to keep in mind principles of adult learning to include autonomy and self-direction, requiring health care professionals to identify their educational needs and plan to meet individual learning objectives. Planning for effective CME should focus on health care professionals expressed individual learning needs and outcomes rather than focusing only on time allocation and budget. We therefore recommend regular effective CME methods for health professionals, on MHCA, with emphasis on selected MHCA forms, namely, urgent application with the medical certificate, voluntary application with a medical recommendation, the reception order and its application, viewed as relevant for admission in district hospitals. Due to insignificant change on the performance of MHCA usages between three and six months following the CME, it may be necessary to consider running such CME twice a year for sustainable effect throughout any year.

## Conclusion

This study was about auditing the use of MHCA forms by health professionals in three periods. It found that the use of required MHCA forms which guarantee lawful admissions of the patients in psychiatric unit was inadequate at LIIMH before CME. An increase in number of MCHA forms from periods CME-0, CME-3, and CME-6 with no statistical difference between the last two periods was noted. Not all MHCA forms were applicable for use in district hospital. We therefore recommend regular effective CME methods for health professionals, on MHCA, with emphasis on selected MHCA forms. Twice a year is advisable for sustainable effect throughout any given year.

### What is known about this topic

- Mental health is an increasing public health issue globally;
- Mental health care act (MHCA) protects the right of patients with mental illnesses as well as health professionals who are providing care;
- Shortcoming encountered in the implementation on mental health in primary care setting can be overcome by the training of health professionals through continuous medical education (CME); effective CME is not to be opportunistic, but regular and continuous.

### What this study adds

- Where many MHCA forms are available for use, mastery of limited numbers of MHCA forms may promote implementation of mental health in primary care;
- No difference is observed in performance following CME between 3 and 6 months;
- Six months could be reasonable interval for sustained retained behavior/skill after a CME.
